# Patient- and population-level health consequences of discontinuing antiretroviral therapy in settings with inadequate HIV treatment availability

**DOI:** 10.1186/1478-7547-10-12

**Published:** 2012-09-19

**Authors:** April D Kimmel, Stephen C Resch, Xavier Anglaret, Norman Daniels, Sue J Goldie, Christine Danel, Angela Y Wong, Kenneth A Freedberg, Milton C Weinstein

**Affiliations:** 1 Department of Healthcare Policy and Research, Virginia Commonwealth University School of Medicine, Richmond, VA, 23298, USA; 2 Weill Cornell Medical College, New York, USA; 3 Harvard School of Public Health, Boston, USA; 4 Harvard Medical School, Boston, USA; 5 INSERM Unité 897, Centre de Recherche “Epidémiologie et Biostatistique” and Université Victor Segalen Bordeaux 2, Bordeaux, France; 6 Programme PAC-CI, Abidjan, Côte d’Ivoire; 7 Massachusetts General Hospital, Boston, USA

**Keywords:** HIV, AIDS, Antiretroviral therapy, ART, Discontinuation, Population health, Ethics, Limited resources

## Abstract

**Background:**

In resource-limited settings, HIV budgets are flattening or decreasing. A policy of discontinuing antiretroviral therapy (ART) after HIV treatment failure was modeled to highlight trade-offs among competing policy goals of optimizing individual and population health outcomes.

**Methods:**

In settings with two available ART regimens, we assessed two strategies: (1) continue ART after second-line failure (Status Quo) and (2) discontinue ART after second-line failure (Alternative). A computer model simulated outcomes for a single cohort of newly detected, HIV-infected individuals. Projections were fed into a population-level model allowing multiple cohorts to compete for ART with constraints on treatment capacity. In the Alternative strategy, discontinuation of second-line ART occurred upon detection of antiretroviral failure, specified by WHO guidelines. Those discontinuing failed ART experienced an increased risk of AIDS-related mortality compared to those continuing ART.

**Results:**

At the population level, the Alternative strategy increased the mean number initiating ART annually by 1,100 individuals (+18.7%) to 6,980 compared to the Status Quo. More individuals initiating ART under the Alternative strategy increased total life-years by 15,000 (+2.8%) to 555,000, compared to the Status Quo. Although more individuals received treatment under the Alternative strategy, life expectancy for those treated decreased by 0.7 years (−8.0%) to 8.1 years compared to the Status Quo. In a cohort of treated patients only, 600 more individuals (+27.1%) died by 5 years under the Alternative strategy compared to the Status Quo. Results were sensitive to the timing of detection of ART failure, number of ART regimens, and treatment capacity. Although we believe the results robust in the short-term, this analysis reflects settings where HIV case detection occurs late in the disease course and treatment capacity and the incidence of newly detected patients are stable.

**Conclusions:**

In settings with inadequate HIV treatment availability, trade-offs emerge between maximizing outcomes for individual patients already on treatment and ensuring access to treatment for all people who may benefit. While individuals may derive some benefit from ART even after virologic failure, the aggregate public health benefit is maximized by providing effective therapy to the greatest number of people. These trade-offs should be explicit and transparent in antiretroviral policy decisions.

## Background

While international initiatives to combat HIV have facilitated major increases in antiretroviral therapy (ART) availability, coverage remains limited [1]. The treatment gap relates to inadequate HIV detection and linkage to care [2], as well as drug stock-outs, funding constraints, and staff and space shortages, contributing to treatment suspensions and waiting lists [3-5]. These obstacles persist when international HIV treatment guidelines call for earlier ART initiation and consideration of additional antiretroviral regimens, which suggest an increasing demand for ART [6]. Changing political priorities and the global financial crisis have also jeopardized external financial commitments to HIV treatment and care [7].

Understanding the range of different treatment alternatives, as well as their associated benefits, costs, and uncertainty, can make trade-offs in clinical policy decisions more explicit. To understand the implications of one area — ART after treatment failure — our objective was to assess a policy of ART discontinuation after failure by creating a stylized depiction of antiretroviral therapy allocation. In so doing, we aimed to highlight trade-offs among competing policy goals of optimizing health outcomes for treated patients, health outcomes for treated and untreated patients, and the number receiving treatment when treatment availability is inadequate.

## Methods

### Overview

This analysis relied on a two-stage modeling approach. We first used a computer model of HIV disease to simulate health outcomes for a cohort of newly detected, HIV-infected individuals in the absence of treatment constraints. We then used these estimates as inputs to a population-level model that allocated treatment across multiple cohorts of newly detected, HIV-infected individuals when treatment capacity was limited. Clinical data were from clinical trials and cohort studies in Côte d’Ivoire, West Africa [8-10]. We evaluated strategies for discontinuing ART (discontinue or not) according to life expectancy, averaged across multiple cohorts of detected HIV-infected individuals. Other performance measures included the mean number initiating treatment annually, mean time on treatment, and mean number alive annually. We conducted sensitivity analyses to examine how key variables and assumptions influenced results.

### Strategies

We evaluated two treatment strategies: (1) continue ART after second-line ART failure (Status Quo), and (2) discontinue ART after second-line ART failure (Alternative). Both strategies include treated as well as untreated, HIV-infected individuals.

In defining the strategies, we made several assumptions. First, all individuals receive two sequential antiretroviral regimens and treatment efficacy is fixed over time [6]. Second, individuals receive semi-annual clinical and immunologic monitoring to assess treatment response, and have quarterly clinic visits [6]. Third, in accordance with WHO guidelines and consistent with clinical care in many resource-limited settings, immunologic and clinical criteria are used to initiate ART, diagnose ART failure, and inform decisions related to regimen switching including, if applicable, discontinuation after second-line failure. ART failure criteria are defined as an observed 50% decrease in peak on-treatment CD4 count, CD4 count <100 μL, CD4 count below pre-ART nadir, or a new WHO stage III/IV event, excluding tuberculosis and severe bacterial infections [6].

### Models

#### Individual-level model

We used a previously described individual-level simulation model (Cost Effectiveness of Preventing AIDS Complications-International) to project strategy-specific life expectancy, the fraction of a cohort receiving treatment annually, and the fraction surviving each year in a single cohort [11-14]. The model simulates a cohort of individual patients whose clinical course is tracked from model entry until death. Disease progression is a function of HIV RNA level, which determines the rate of CD4 count decline and, in turn, the risk of specific opportunistic infections and death [15]. For patients receiving ART, virologically suppressed patients experience HIV RNA decreases and CD4 count increases, with a decrease in CD4-specific morbidity and mortality; a fraction of those virologically suppressed experience no CD4 increase in response to treatment [16]. Patients with virologic failure but who remain on ART have an independent reduction in AIDS-related mortality compared to those not receiving ART [17]. Patients on ART who become lost to follow-up experience an initial period of increased risk of morbidity and mortality compared to those not lost; those who are lost and experience a WHO stage IV event may re-enter care [18].

#### Population-level model

We used estimates from the individual-level simulation model as inputs to a population-level linear programming model to estimate health outcomes in the total HIV-infected population, including untreated patients, when there are real-world constraints on treatment capacity. The population-level model seeks to maximize accumulated life-years for multiple cohorts of newly detected, HIV-infected individuals. The model chooses an optimal fraction of each cohort to receive ART, subject to constraints on treatment capacity. The constraint on capacity is in the form of treatment slots, defined as the number treated annually, which serves as a proxy for the many constraints (e.g., financing, human resource capacity, health and social service capacity, and personnel affordability) faced by public sector ART programs. Time on ART per treated patient determines consumption of these slots. The model is specified formally in the Additional file 1.

### Analysis

We compared the relative performance of strategies using long-term health outcomes, or life expectancy per cohort. We also evaluated total life-years accumulated across cohorts and, to address a competing policy objective of treating as many persons as possible, the mean number of individuals initiating treatment annually. In addition, we examined ART coverage, defined as the ratio of the number receiving treatment annually to the number qualifying for treatment annually, and strategy-specific survival over time. We evaluated each outcome over a 5-year analytic time horizon.

### Input parameters

#### Individual-level model

Cohort characteristics and natural history. Data were from trials and cohort studies conducted in Côte d’Ivoire by the Programme PAC-CI. Initial distributions of age, sex, and CD4 count were derived from the ACONDA cohort, an observational cohort of HIV-infected adults and a continuation of the ANRS 1203 Cotrame cohort study in Abidjan (Table 1) [18,19]. Incidence of opportunistic infections, HIV-related mortality, and efficacy and toxicity of cotrimoxazole prophylaxis were from ANRS 059 trial data, as well as from the ANRS 1203 and 1220 study cohorts [9,10,20]. Risk of non-HIV–related mortality was from country-specific life tables for Côte d’Ivoire [21]. 

**Table 1:** Selected data for the individual-level model

**Variable**	**Base case value**	**Reference(s)**
Initial cohort characteristics		
Mean age (SD) (yrs)	36.9 (9.2)	Touré et al. [18]
Gender distribution	70% female	Touré et al. [18]
Mean CD4 count (SD) (cells/μL)	140 (116)	Touré et al. [18]
Median HIV RNA (IQR) (log_10_ copies/mL)	5.3 (4.8–5.8)	Seyler et al. [19]
First- and second-line antiretroviral efficacy*		
HIV RNA suppression at 24 weeks	80.2%	Messou et al. [22]
CD4 count increase at 24 weeks (cells/μL)†	+152	Messou et al. [22]
Probability of discordant response	5%	Grabar et al. [16]
Loss to follow-up		
18-month cumulative loss to follow-up	15%	Touré et al. [18]
Probability of returning to care if WHO stage IV event	50%	Assumption

*SD* standard deviation, *WHO* World Health Organization, *NNRTI* non-nucleoside reverse transcriptase inhibitor, *PI* protease inhibitor, *IQR* interquartile range.

*First-line antiretroviral therapy (ART) efficacy data were derived from the ACONDA cohort, in which 52% received an initial ART regimen of stavudine, lamivudine, and nevirapine; 22% received stavudine, lamivudine, and efavirenz; and 20% received zidovudine, lamivudine, and efavirenz (with the remaining 6% receiving other regimens). We assumed a dosing scheduled in accordance with WHO recommendations — 300 mg once daily (zidovudine), 150 mg twice daily (lamivudine), 30 mg twice daily (stavudine), 600 mg once daily (efavirenz), and 200 mg once daily (nevirapine). In the absence of data, we assumed that second-line ART suppression rates were identical to that for first-line ART.

†For first-line ART, CD4 count increases were 76 (standard deviation (SD) 19) cells/μL per month for months 1–2 and 4 (SD 1) cells/μL per month thereafter. We assumed similar CD4 response for second-line ART.

ART. Effectiveness of non-nucleoside reverse transcriptase inhibitor-based first-line ART was from a cohort of treatment-naïve patients in Abidjan (at 24 weeks, 80.2% of patients HIV RNA suppressed to ≤300 copies/mL and median CD4 count increase of 152 cells/μL (IQR 64/μL, 201/μL)) [22]. We assumed that the effectiveness of protease inhibitor- (PI-) based second-line ART, and, in sensitivity analysis, of a third-line regimen was similar to that of first-line ART. Five percent of those virologically suppressed experienced a discordant response, or no immunologic response to ART [16]. In addition, 15% of patients receiving ART were lost to follow-up by 18 months [18]; among those lost, we assumed 50% of those who later experienced a WHO stage IV event re-entered care.

#### Population-level model

The individual-level model produced several projections used as inputs to the population-level model. These included: (1) strategy-specific life expectancy, and (2) the number receiving ART annually, which was used to derive the strategy-specific annual probability of receiving ART and cohort and strategy-specific annual treatment need.

For parameters regarding the number of newly detected patients annually and total treatment slots, we drew upon projections from the UNAIDS Spectrum model and recent data on antiretroviral coverage, both for Côte d’Ivoire. Spectrum model estimates for the number needing first-line ART annually (30,000) were used as a proxy for the number newly detected each year, since most HIV-infected individuals present with relatively advanced disease and are, therefore, eligible for ART upon detection [23]. Estimates of the total number of treatment slots at any one time (50,000) were based on recent UNAIDS data on the total number receiving ART in Côte d’Ivoire [24].

## Results

### Base case

For a cohort of newly detected HIV-infected individuals with mean CD4 140 cells/μL and no access to ART, life expectancy was 1.9 years. For treated individuals only, compared to the Status Quo, the Alternative strategy decreased life expectancy from 8.8 to 8.1 years (Table 2). Under the Alternative strategy, treated individuals spent 6.3 years on ART, or 1.1 years less than under the Status Quo.

**Table 2:** Base case results: individual- and population-level antiretroviral health benefits in a setting with inadequate treatment availability

	**Treated individuals only**		**Treated and untreated individuals†**			
Strategy*	**Life expectancy (Years)**	**Mean time on treatment (Years)**	**Mean number initiating treatment annually**	**Life expectancy (Years)**	**Total life-years (Years)**	**Mean annual treatment coverage‡ (%)**
Status Quo	8.8	7.4	5,880	3.6	540,000	24.4
Alternative	8.1	6.3	6,980	3.7	555,000	29.0

*In the Status Quo, antiretroviral therapy (ART) is never discontinued. In the Alternative strategy, ART is discontinued when second-line ART failure is observed. In the base case, ART failure is defined as a 50% decrease in peak on-treatment CD4 count, CD4 count <100 cells/μL, CD4 count below pre-ART nadir, or a WHO stage III/IV event, excluding tuberculosis and severe bacterial infections [6]. On average, individuals who received no treatment lived approximately 1.9 years.

†Results are presented for a 5-year analytic time horizon for a cohort of 30,000 newly detected HIV-infected individuals entering care annually.

‡Treatment coverage is defined as the ratio of the number receiving treatment annually to the number qualifying for treatment annually.

Mean time on ART among treated individuals influenced the number initiating treatment annually when multiple cohorts of newly detected individuals competed for treatment slots (Table 2). Of the 150,000 identified over 5 years, 5,880 individuals on average initiated ART annually under the Status Quo, resulting in life expectancy per cohort of 3.6 years; 540,000 accumulated life-years (3.6 years x 30,000 individuals/cohort x 5 cohorts = 540,000 life-years); and ART coverage of 24.4%. Compared to the Status Quo, the Alternative strategy increased the mean number initiating ART annually by 1,100 to 6,980, which increased life expectancy per cohort by 0.1 years to 3.7 years; total life-years accumulated by 15,000 to 555,000 life-years; and ART coverage by 18.9% to 29.0%.

**Figure 1:**
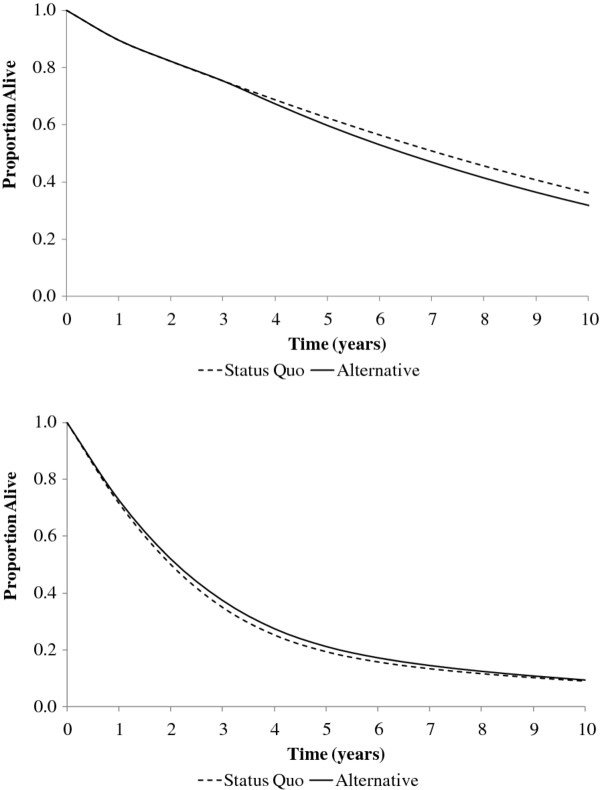
**Survival, by strategy, for a single cohort of treated individuals only (Upper Panel) and a single cohort of both treated and untreated individuals (Lower Panel) when treatment slots are limited to 50,000.** On the *x-*axis is time; on the *y-*axis is the proportion alive. By 5 years, survival among a single cohort of treated individuals in the Status Quo exceeds survival among treated individuals in the Alternative strategy. In contrast, for a single cohort of treated and untreated individuals followed over 5 years, survival under the Alternative strategy exceeds survival under the Status Quo.

To gain further insight into the health consequences of each strategy, we also evaluated strategy-specific survival (Figure 1). At 5 years, 62% survived under the Status Quo compared to 59% under the Alternative strategy. Of the 5,880 individuals initiating ART annually under the Status Quo, 2,210 died over 5 years, compared to 2,810 among the 6,980 individuals initiating ART under the Alternative strategy. This resulted in 600 more deaths over 5 years for a single cohort of treated individuals under the Alternative strategy compared to the Status Quo. For a single cohort of treated and untreated individuals, there were 1,120 fewer deaths under the Alternative strategy compared to the Status Quo over a 5-year period.

### Sensitivity analysis

**Figure 2:**
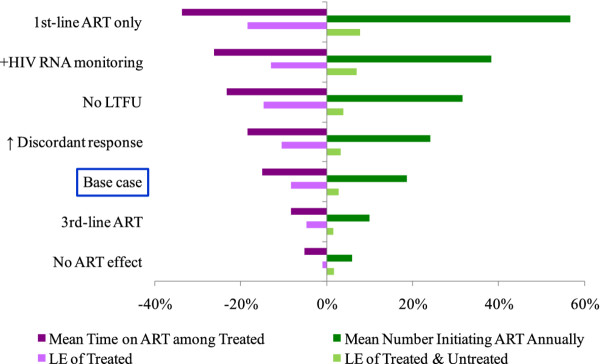
**Sensitivity analysis: percent difference of the Alternative strategy compared to the Status Quo for two health outcomes.** Variation in health outcomes is shown on the horizontal axis and results from changes in select individual-level model parameters, which are listed on the vertical axis. To the left of the origin (i.e., 0%) is the percent difference of the Alternative strategy compared to the Status Quo regarding mean time on treatment among treated individuals (dark purple) and life expectancy of treated individuals (light purple). To the right of the origin is the percent difference of the Alternative strategy compared to the Status Quo regarding the mean number initiating antiretroviral therapy (ART) annually (dark green) and life expectancy of both treated and untreated individuals (light green). “+HIV RNA monitoring” refers to the addition of both HIV RNA monitoring to base case assumptions. “↑ Discordant response” indicates an increase in the fraction of discordant responses to ART (i.e., no immunologic response to ART among those virologically suppressed) from 5% to 19.1%. “ART effect” refers to the independent effect of ART on AIDS-related mortality. The percent difference in life expectancy among treated and untreated individuals for the Alternative strategy compared to the Status Quo is less than among treated individuals only, a sub-population in this analysis. Therefore, the percent difference in life expectancy at the population level serves as a conservative estimate of the public health benefit of the Alternative strategy. ART: antiretroviral therapy; LTFU: loss to follow-up; LE: life expectancy.

We evaluated the impact of uncertain parameters and assumptions on the results (Figure 2). Among treated and untreated individuals, the advantage of the Alternative strategy compared to the Status Quo was most sensitive to: timing of detecting ART failure, the number of available ART regimens, and the independent effect of ART on AIDS-related mortality. The advantage of the Alternative strategy was less sensitive to timing of ART initiation (e.g., later ART initiation at CD4 <200 cells/μL as recommended in the 2006 WHO guidelines [25]), second-line virologic suppression, probability of later virologic failure after initial suppression, and increases in the proportion of discordant response to ART. However, while results were sensitive to large increases (or decreases) in 18-month loss to follow-up as shown in Figure 2, they were less sensitive to small variations in this value. For example, we evaluated a scenario in which 18-month loss to follow-up increased 10%, a value in line with data on loss to follow-up from some higher prevalence settings [26]. Here, among treated individuals only, the Alternative strategy decreased life expectancy from 8.5 to 7.8 years (−8.2%) compared to the Status Quo. On average, treated individuals received ART for 6.0 years, or 0.9 fewer years on ART (−15.7%) compared to the Status Quo. This increased the number of ART-eligible individuals initiating treatment by 1,130 to 7,300 (+18.3%) and increased accumulated life-years by 10,000 to 560,000 life-years (+1.9%) (see Additional file 1). In contrast, results were less sensitive to assumptions made about returning to care once an individual is lost from care, including if we specified that individuals lost who later had a WHO stage IV event did not re-enter care (see Additional file 1). 

Sensitivity analyses also suggested that the population-level advantage of the Alternative strategy decreased as the number of antiretroviral regimens increased. For example, if only a single ART regimen were available, as might occur if second-line options are severely limited [27], treated individuals only lived 1.3 fewer years (−17.8%) and spent 2.1 fewer years on ART (−33.9%) under the Alternative strategy compared to the Status Quo, resulting in 4,020 more individuals initiating ART annually (+56.7%) and 42,000 more accumulated life-years at the population level (+7.9%). If as many as three ART regimens were available, treated individuals only lived 0.5 fewer years (−5.3%) and spent 0.6 fewer years on ART under the Alternative strategy compared to the Status Quo (−7.5%), resulting in 540 more individuals initiating ART annually (+10%) and 8,000 more accumulated life-years at the population level (+1.5%) (see Figure 2 and Additional file 1).

Assumptions about treatment capacity most influenced the health benefits for treated and untreated individuals. For every 5,000 additional treatment slots available, life expectancy per cohort increased approximately 2 life-months; this gain was accompanied by increases in the number initiating ART annually, total accumulated life-years, and ART coverage. The Alternative strategy maximized life expectancy per cohort and the number initiating ART annually unless treatment capacity exceeded 175,000 slots (corresponding to antiretroviral coverage of approximately 85%) or, alternatively, when the annual incidence of newly detected cases was <10,000 per year. In these two instances, the Status Quo maximized life expectancy per cohort.

**Figure 3:**
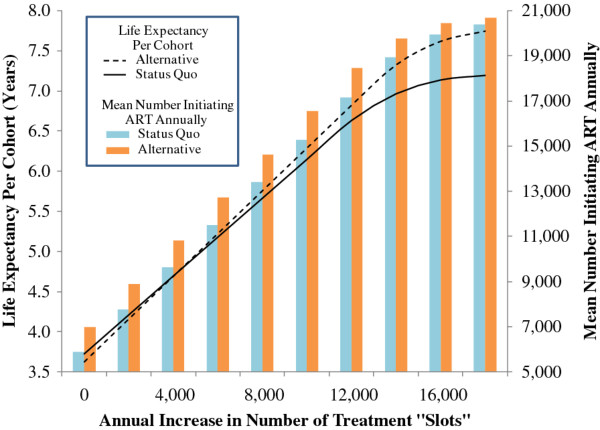
**Sensitivity analysis: impact of increasing treatment capacity over time.** This figure illustrates the impact on two health outcomes of increasing treatment capacity over time, while holding the incidence of newly detected patients constant. On the horizontal axis is the annual increase in the number of treatment “slots”. Variation in the number of additional treatment slots annually impacts life expectancy per cohort (left vertical axis) and the mean number initiating antiretroviral therapy (ART) annually (right vertical axis). Below an annual increase of approximately 4,000 treatment slots, life expectancy per cohort under the Alternative strategy (dashed line) exceeds life expectancy per cohort under the Status Quo (solid line). Above this threshold, the relationship reverses and life expectancy per cohort under the Status Quo exceeds that under the Alternative strategy. In contrast, regardless of the annual increase in treatment capacity, the mean number initiating ART annually under the Alternative strategy (orange) always is greater than the mean number initiating ART annually under the Status Quo (blue). However, the difference in the mean number initiating ART annually between the two strategies begins to decrease when treatment capacity exceeds 62,000 treatment slots, or an approximate 25% increase, at any one time.

Finally, to gain insight into real-world variation in treatment supply and demand, we relaxed the steady-state assumption of constant treatment capacity and allowed capacity to increase over time while holding constant the number of newly detected patients per cohort (Figure 3). At or below 4,000 additional treatment slots annually, the Alternative strategy remained optimal. Above this threshold, life expectancy per cohort was maximized by the Status Quo.

## Discussion

With evidence of recent funding shortfalls, continued obstacles to treatment provision, and flattening donor funding across resource-limited settings, we sought to assess the consequences and identify the trade-offs associated with one HIV treatment policy decision — antiretroviral discontinuation after treatment failure. To do so, we evaluated life expectancy for individual treated patients; life expectancy for an HIV-infected population, including treated and untreated individuals; and the number receiving treatment when discontinuing ART compared to the current standard of care (i.e., lifelong ART).

Results confirm that among treated individuals only, treatment discontinuation results in lower life expectancy and decreased treatment resource consumption. Among both treated and untreated individuals, however, discontinuing treatment among those who have failed it increases the total number of individuals receiving therapy, and thereby increases life expectancy per cohort among all newly detected HIV-infected individuals. This relationship holds across a variety of different assumptions, including many levels of treatment capacity, ART initiation criteria, number of treatment regimens, and timing of antiretroviral failure detection.

This research highlights concerns regarding the responsibility health care providers feel to patients directly under their care compared to those in the wider community. In this analysis, more deaths occur over the analytic time horizon for treated individuals under the Status Quo compared to the Alternative strategy. This finding underscores both clinical and ethical concerns in resource-limited settings. First, some fraction of deaths among treated individuals are deaths which would likely be avoidable with less misclassification of ART failure [28]. Out of concern for and responsibility to patients under their care, many health care providers would choose not to implement, or even consider, a treatment discontinuation policy unless a treated patient was not receiving any ART-related health benefits. In settings where HIV RNA and/or genotype tests are not available, a discontinuation policy implies ART may be withdrawn while it is still effective, due to discordance between immunologic and virologic failure and uncertainty regarding the underlying cause of virologic failure (e.g., non-adherence). Given the lack of adequate diagnostic surrogates for both HIV RNA and genotype tests, a discontinuation policy might only be feasible in settings with access to these tests.

Further, the increased number of deaths under the Alternative strategy, compared to the Status Quo, are deaths for which a health care provider would feel immediately responsible. Indeed, health care providers, as providers, would oppose a policy of treatment discontinuation because of their sense of obligation to offer the best treatment to patients under their care. This opposition might well continue even if another person could potentially benefit more from the discontinued treatment. Therefore, even if misclassification of antiretroviral failure were eliminated, concerns about patient abandonment and, for physicians, mindfulness of the Hippocratic Oath may well persist even if fewer deaths occurred in the overall HIV-infected population under the Alternative strategy compared to the Status Quo. For these reasons, when conflict exists between the health outcomes of individual patients in care and those of the larger community, it is important for clinicians and public health authorities to work closely together, so that policy discussions consider both the best interests of individual patients as well as the wider population.

Increasing treatment capacity over time highlights trade-offs associated with the goals of different treatment policies. With more modest capacity increases over time, discontinuing treatment not only maximizes life expectancy per cohort, but also allows more individuals to receive treatment, improving both efficiency and equity. With greater capacity increases over time, however, the Status Quo (i.e., not discontinuing treatment) maximizes life expectancy per cohort, yet does not allow more individuals to receive treatment. In this case, the greater health benefits associated with lifelong treatment outweigh the increased numbers of individuals who initiate, but later discontinue, ART. Therefore, efficiency improves but at the expense of equity. This illustrates the tension, given a specific treatment capacity, between keeping individuals on treatment longer and treating more people. While this model was developed to address efficiency concerns and maximize life expectancy per cohort, instances could arise in which concerns about equity override efficiency. This might occur, for example, if society values certain distributions of services (i.e., more individuals receiving ART under a discontinuation policy) more highly than overall population benefit resulting from a particular treatment policy.

While obstacles exist to implementing an antiretroviral discontinuation policy when treatment failure has occurred and treatment availability is inadequate, political challenges may limit the broader discussion and formulation of clearly articulated prioritization policies. Indeed, the adoption of such policies can be influenced by the prospect of far-reaching influence (e.g., a majority vote); targeted impact (e.g., interest groups, such as funding agencies, health authorities, or activists); political reward (e.g., media or public opinion polling, legacy, or mandates); or individual reward (e.g., seeking policies that will increase personal advantage at the expense of societal benefit), all of which policy makers may consider in the policy acceptance and/or implementation process [29-31].

While this study suggests greater public health benefits could be realized through implementation of the Alternative strategy when treatment availability is inadequate, additional challenges exist regarding explicit priority setting even when resources are severely constrained. Given the complexity of resource allocation decisions, Mechanic, while acknowledging the role of explicit rationing decisions in providing a framework for medical care, has cautioned against formal rationing in the doctor-patient relationship and the process of care provision [32]. He argues that the process of providing medical care develops both personally and iteratively, relying on patient trust and diverse preferences rather than the implementation of rigid standards and a one-size-fits-all approach. These personal relationships result in exceptions to the rule in the process of providing care. However, explicit rules or standards of care may lack flexibility in their implementation, may be subject to political manipulation, and may lag behind the changing reality of clinical care and uncertainty in the evidence base. In the current climate, he later argues, these challenges can begin to be met through several mechanisms, including patient advocacy within frameworks of procedural justice and fair process, expanded responsibility for population health, more collaborative and participatory partnerships with patients, and practicing of medicine that has transparent rationales and a clear evidence base [33]. Ham and Coulter propose an integrated approach to priority setting that they believe captures the complexity of allocation decisions in practice [34]. Recognizing that while explicit priority setting can enhance fairness, political accountability, and transparency, they cite the need for an improved informational and institutional base to inform decision making. Continued quantification and articulation of priority decisions by experts complemented by strengthened institutions that can better incorporate input from both experts and, in particular, the public are recommended [34].

This study has several limitations. First, we assume the number of treatment regimens available and antiretroviral regimen efficacy is fixed over time. We found, however, that the results hold with fewer as well as more antiretroviral regimens, though the impact is mitigated if more regimens are available. Second, similar to treatment slots, time on treatment was chosen as a proxy for the consumption of HIV treatment resources. Third, this analysis relied mainly on data available from Côte d’Ivoire, which may limit the generalizability of results. However, parameter estimates generally fall within the confidence intervals of data from other resource-limited settings, including first- and second-line ART effectiveness at 24 weeks and 18-month loss from care [27,35-38]. While this allows drawing of broad policy conclusions about antiretroviral discontinuation after treatment failure, context-specific analyses that rely on sound, local data should be conducted in settings where complete treatment availability may be inadequate.

Fourth, while the population model implicitly includes costs in the treatment slot constraint, the analysis does not explicitly account for ART costs, a key driver of policy decisions in this context [39]. However, it is unlikely that policy conclusions would change were ART and opportunistic infection costs included in the analysis. This is because the higher cost of continued second-line ART after first-line antiretroviral failure under the Status Quo strategy would outweigh the combined costs of: (a) newly enrolled patients on less expensive first-line ART, including transaction or start-up costs associated with treatment enrollment, and (b) treatment for opportunistic infections among those discontinuing treatment under the Alternative strategy. Therefore, at the population level, the comparative advantage of the Alternative strategy compared to the Status Quo would remain similar or be strengthened if costs related to treatment and care were included.

Fifth, in the population model, we assume a steady state situation, in which there is a constant incidence of newly detected cases, distribution of patient characteristics of detected cases, and availability of treatment. In the short-term, as in the 5-year time horizon in this analysis, steady state assumptions may be plausible. For example, recent reports suggest that the HIV epidemic has stabilized in some sub-Saharan African countries [40], indicating that HIV incidence, and potentially, the incidence of newly detected patients, could remain relatively stable in the short-term in some settings. However, prevention effects of ART, which evidence shows decrease the risk of HIV transmission [41,42], may result in lower numbers of newly HIV-infected individuals annually, lower numbers of newly detected cases annually, and potentially decreased demand for HIV treatment in the longer term. In addition, a larger fraction of treated individuals would have effective viral suppression under the Alternative strategy, which would increase the population health advantage of the Alternative strategy compared to the Status Quo. In the current economic climate of flat or decreasing HIV treatment budgets, it is also reasonable to assume that capacity, in the form of treatment slots, might remain constant over a fixed time horizon. However, if voluntary counseling and testing efforts and/or treatment scale-up efforts continue, it is unlikely that treatment demand, in the form of newly detected patients, and treatment capacity will remain fixed over time.

Finally, in the population-level model, we assume individuals could initiate ART only upon HIV case detection. In settings where HIV case detection occurs late in the course of disease (typically CD4 <200 cells/μL) [43], as in this analysis, the vast majority of individuals entering a treatment program are already ART eligible. Thus, precluding later treatment initiation once the eligibility criterion is met has little effect on the results. If individuals eligible to initiate treatment upon detection, but for whom no treatment slot was available were able to subsequently initiate ART, the health benefit achieved by these individuals would likely be less than among individuals initiating ART immediately upon detection. Therefore, in this model, which maximizes life expectancy per cohort, the remaining fraction of a previous cohort would not be selected to receive ART if competing with newly detected individuals who could achieve higher life expectancy.

## Conclusions

As obstacles to providing HIV treatment persist in resource-limited settings, questions regarding resource allocation and priority setting necessarily emerge. Assessment of antiretroviral discontinuation policies can highlight the trade-offs associated with policy goals of maximizing life expectancy of treated individuals only, life expectancy of the entire population, and the number of individuals receiving treatment. While individuals receiving ART may continue to derive some treatment benefit even after virologic failure, the aggregate public health benefit is maximized by providing effective therapy to the greatest number of people. Individual- and population-level health trade-offs should be debated and discussed, with these trade-offs made explicit and more transparent in treatment policy decisions.

## Endnote

Preliminary results for this manuscript were presented in part at the Annual Meeting of the Society for Medical Decision Making [abstract 5218], October 24–27, 2010, Toronto, Canada.

## Abbreviation

ART: Antiretroviral Therapy.

## Competing interests

The authors declare that they have no competing interests.

## Authors’ contributions

All authors (ADK, SCR, XA, ND, SJG, CD, AW, KAF, MCW) agree with the results and conclusions of the manuscript. ADK, SCR, SJG, and MCW designed the study. ADK, SCR, and AW analyzed the data. XA, CD, and ADK collected the data. ADK, SCR, ND, KAF, and MCW contributed to analysis and interpretation of results. ADK wrote the first draft of the paper. SCR, XA, ND, SJG, CD, KAF, and MCW contributed to writing the manuscript. All authors read and approved the final manuscript.

## Supplementary Material

Additional file 1 Supplemental methods and results. Click here for file

## References

[B1] World Health Organization Towards universal access: scaling up priority HIV/AIDS interventions in the health sector Progress report 2010 2010 Accessed September 15, 2012, at http://whqlibdoc.who.int/publications/2010/9789241500395_eng.pdf

[B2] Matovu JK Makumbi FE Expanding access to voluntary HIV counselling and testing in sub-Saharan Africa: alternative approaches for improving uptake, 2001–2007 Trop Med Int Health 2007 12 1315 1322 10.1111/j.1365-3156.2007.01923.x 17949401

[B3] Muhamadi L Xavier N Mbona TN Wabwire-Mangen F Anna-Mia E Stefan P George P Inadequate pre-antiretroviral care, stock-out of antiretroviral drugs and stigma: Policy challenges/bottlenecks to the new WHO recommendations for earlier initiation of antiretroviral therapy (CD < 350 cells/muL) in eastern Uganda Health Policy 2010 97 187 194 10.1016/j.healthpol.2010.06.003 20615573

[B4] Posse M Meheus F van Asten H van der Ven A Baltussen R Barriers to access to antiretroviral treatment in developing countries: a review Trop Med Int Health 2008 13 904 913 10.1111/j.1365-3156.2008.02091.x 18466183

[B5] Wasswa H Ugandan hospitals ration AIDS treatment as antiretrovirals start to run out BMJ 2010 341 c3900 10.1136/bmj.c3900 20647289

[B6] World Health Organization Antiretroviral therapy for HIV infection in adults and adolescents: recommendations for a public health approach 2010 revision 2010 Accessed September 15, 2012, at http://whqlibdoc.who.int/publications/2010/9789241599764_eng.pdf 23741771

[B7] Kates J Boortz K Lief E Avila C Gobet B Financing the response to AIDS in low- and middle-income countries: International assistance from the G8, European Commission and other donor governments in 2009 2010 Accessed September 15, 2012, at http://www.kff.org/hivaids/upload/7347-052.pdf

[B8] Anglaret X Chene G Attia A Touré S Lafont S Combe P Manlan K N’Dri-Yoman T Salamon R Early chemoprophylaxis with trimethoprim-sulphamethoxazole for HIV-1-infected adults in Abidjan, Côte d’Ivoire: a randomised trial Cotrimo-CI Study Group Lancet 1999 353 1463 1468 10.1016/S0140-6736(98)07399-1 10232311

[B9] Minga A Danel C Abo Y Dohoun L Bonard D Coulibaly A Duvignac J Dabis F Salamon R Anglaret X Progression to WHO criteria for antiretroviral therapy in a 7-year cohort of adult HIV-1 seroconverters in Abidjan, Côte d’Ivoire Bull World Health Organ 2007 85 116 123 10.2471/BLT.06.032292 17308732 PMC2636271

[B10] Seyler C Messou E Gabillard D Inwoley A Alioum A Anglaret X Morbidity before and after HAART initiation in Sub-Saharan African HIV-infected adults: a recurrent event analysis AIDS Res Hum Retroviruses 2007 23 1338 1347 10.1089/aid.2006.0308 18184075

[B11] Goldie SJ Yazdanpanah Y Losina E Weinstein MC Anglaret X Walensky RP Hsu HE Kimmel A Holmes C Kaplan JE Freedberg KA Cost-effectiveness of HIV treatment in resource-poor settings–the case of Côte d’Ivoire N Engl J Med 2006 355 1141 1153 10.1056/NEJMsa060247 16971720

[B12] Walensky RP Weinstein MC Yazdanpanah Y Losina E Mercincavage LM Touré S Divi N Anglaret X Goldie SJ Freedberg KA HIV drug resistance surveillance for prioritizing treatment in resource-limited settings AIDS 2007 21 973 982 10.1097/QAD.0b013e328011ec53 17457091 PMC2367006

[B13] Kimmel AD Weinstein MC Anglaret X Goldie SJ Losina E Yazdanpanah Y Messou E Cotich KL Walensky RP Freedberg KA Laboratory monitoring to guide switching antiretroviral therapy in resource-limited settings: clinical benefits and cost-effectiveness J Acquir Immune Defic Syndr 2010 54 258 268 10.1097/QAI.0b013e3181d0db97 20404739 PMC3174771

[B14] Losina E Touré H Uhler LM Anglaret X Paltiel AD Balestre E Walensky RP Messou E Weinstein MC Dabis F Freedberg KA Cost-effectiveness of preventing loss to follow-up in HIV treatment programs: a Côte d’Ivoire appraisal PLoS Med 2009 6 e1000173 10.1371/journal.pmed.1000173 19859538 PMC2762030

[B15] Mellors JW Munoz A Giorgi JV Margolick JB Tassoni CJ Gupta P Kingsley LA Todd JA Saah AJ Detels R Plasma viral load and CD4+ lymphocytes as prognostic markers of HIV-1 infection Ann Intern Med 1997 126 946 954 9182471 10.7326/0003-4819-126-12-199706150-00003

[B16] Grabar S Le Moing V Goujard C Leport C Kazatchkine MD Costagliola D Weiss L Clinical outcome of patients with HIV-1 infection according to immunologic and virologic response after 6 months of highly active antiretroviral therapy Ann Intern Med 2000 133 401 410 10975957 10.7326/0003-4819-133-6-200009190-00007

[B17] Losina E Yazdanpanah Y Deuffic-Burban S Wang B Wolf LL Messou E Gabillard D Seyler C Freedberg KA Anglaret X The independent effect of highly active antiretroviral therapy on severe opportunistic disease incidence and mortality in HIV-infected adults in Côte d’Ivoire Antivir Ther 2007 12 543 551 17668563 PMC3073611

[B18] Touré S Kouadio B Seyler C Traore M Dakoury-Dogbo N Duvignac J Diakite N Karcher S Grundmann C Marlink R Rapid scaling-up of antiretroviral therapy in 10,000 adults in Côte d’Ivoire: 2-year outcomes and determinants AIDS 2008 22 873 882 10.1097/QAD.0b013e3282f768f8 18427206 PMC3921665

[B19] Seyler C Anglaret X Dakoury-Dogbo N Messou E Touré S Danel C Diakite N Daudie A Inwoley A Maurice C Medium-term survival, morbidity and immunovirological evolution in HIV-infected adults receiving antiretroviral therapy, Abidjan, Côte d’Ivoire Antivir Ther 2003 8 385 393 14640385

[B20] El-Sadr WM Lundgren JD Neaton JD Gordin F Abrams D Arduino RC Babiker A Burman W Clumeck N Cohen CJ CD4+ count-guided interruption of antiretroviral treatment N Engl J Med 2006 355 2283 2296 17135583 10.1056/NEJMoa062360

[B21] Lopez AD World Health Organization: World mortality in 2000: life tables for 191 countries 2002 World Health Organization, Geneva

[B22] Messou E Kouakou M Gabillard D Gouessé P Koné M Tchehy T Losina E Freedberg KA Anzian A Toure S Anglaret X Medication possession ratio: predicting and decreasing loss to follow-up in antiretroviral treatment programs in Côte d’Ivoire J Acquir Immune Defic Syndr 2011 57 Suppl 1 S34 S39 21857284 10.1097/QAI.0b013e3182208003 PMC3164962

[B23] Stover J Johnson P Zaba B Zwahlen M Dabis F Ekpini RE The Spectrum projection package: improvements in estimating mortality, ART needs, PMTCT impact and uncertainty bounds Sex Transm Infect 2008 84 Suppl 1 i24 i30 18647862 10.1136/sti.2008.029868 PMC2569834

[B24] UNAIDS/WHO Working Group on Global HIV/AIDS and STI Surveillance Epidemiological fact sheets on HIV and AIDS. core data on epidemiology and response 2008 Côte d’Ivoire update. 2008. Accessed September 15, 2012, at http://apps.who.int/globalatlas/predefinedReports/EFS2008/full/EFS2008_CI.pdf

[B25] World Health Organization Antiretroviral therapy for HIV infection in adults and adolescents in resource-limited settings: towards universal access Recommendations for a public health approach. 2006 revision 2006 Accessed September 15, 2012, at http://www.who.int/hiv/pub/guidelines/artadultguidelines.pdf

[B26] Boulle A Bock P Osler M Cohen K Channing L Hilderbrand K Mothibi E Zweigenthal V Slingers N Cloete K Abdullah F Antiretroviral therapy and early mortality in South Africa Bull World Health Organ 2008 86 678 687 10.2471/BLT.07.045294 18797643 PMC2649489

[B27] Ajose O Mookerjee S Mills EJ Boulle A Ford N Treatment outcomes of patients on second-line antiretroviral therapy in resource-limited settings: a systematic review and meta-analysis AIDS 2012 26 929 938 10.1097/QAD.0b013e328351f5b2 22313953

[B28] Kantor R Diero L Delong A Kamle L Muyonga S Mambo F Walumbe E Emonyi W Chan P Carter EJ Misclassification of first-line antiretroviral treatment failure based on immunological monitoring of HIV infection in resource-limited settings Clin Infect Dis 2009 49 454 462 10.1086/600396 19569972 PMC4859427

[B29] Goddard M Hauck K Smith PC Priority setting in health - a political economy perspective Health Econ Policy Law 2006 1 79 90 18634704 10.1017/S1744133105001040

[B30] McCaughey D Bruning NS Rationality versus reality: the challenges of evidence-based decision making for health policy makers Implement Sci 2010 5 39 10.1186/1748-5908-5-39 20504357 PMC2885987

[B31] Robinson R Limits to rationality: economics, economists and priority setting Health Policy 1999 49 13 26 10.1016/S0168-8510(99)00040-8 10827288

[B32] Mechanic D Muddling through elegantly: finding the proper balance in rationing Health Aff (Millwood) 1997 16 83 92 10.1377/hlthaff.16.5.83 9314678

[B33] Mechanic D Managed care and the imperative for a new professional ethic Health Aff (Millwood) 2000 19 100 111 10.1377/hlthaff.19.5.100 10992657

[B34] Ham C Coulter A Explicit and implicit rationing: taking responsibility and avoiding blame for health care choices J Health Serv Res Policy 2001 6 163 169 10.1258/1355819011927422 11467274

[B35] Fox MP Rosen S Patient retention in antiretroviral therapy programs up to three years on treatment in sub-Saharan Africa, 2007–2009: systematic review Trop Med Int Health 2010 15 Suppl 1 1 15 20586956 10.1111/j.1365-3156.2010.02508.x PMC2948795

[B36] Laurent C Diakhate N Gueye NF Toure MA Sow PS Faye MA Gueye M Laniece I Toure Kane C Liegeois F The Senegalese government’s highly active antiretroviral therapy initiative: an 18-month follow-up study AIDS 2002 16 1363 1370 10.1097/00002030-200207050-00008 12131213

[B37] Laurent C Kouanfack C Koulla-Shiro S Nkoue N Bourgeois A Calmy A Lactuock B Nzeusseu V Mougnutou R Peytavin G Effectiveness and safety of a generic fixed-dose combination of nevirapine, stavudine, and lamivudine in HIV-1-infected adults in Cameroon: open-label multicentre trial Lancet 2004 364 29 34 10.1016/S0140-6736(04)16586-0 15234853

[B38] Hammond R Harry TC Efficacy of antiretroviral therapy in Africa: effect on immunological and virological outcome measures – a meta-analysis Int J STD AIDS 2008 19 291 296 10.1258/ijsa.2007.007248 18482957

[B39] Moszynski P Global Fund suspends new projects until 2014 because of lack of funding BMJ 2011 343 d7755 10.1136/bmj.d7755 22127774

[B40] UNAIDS/WHO AIDS epidemic update 2009 Accessed September 15, 2009, at http://data.unaids.org/pub/Report/2009/JC1700_Epi_Update_2009_en.pdf

[B41] Cohen MS Chen YQ McCauley M Gamble T Hosseinipour MC Kumarasamy N Hakim JG Kumwenda J Grinsztejn B Pilotto JH Prevention of HIV-1 infection with early antiretroviral therapy N Engl J Med 2011 365 493 505 10.1056/NEJMoa1105243 21767103 PMC3200068

[B42] Donnell D Baeten JM Kiarie J Thomas KK Stevens W Cohen CR McIntyre J Lingappa JR Celum C Heterosexual HIV-1 transmission after initiation of antiretroviral therapy: a prospective cohort analysis Lancet 2010 375 2092 2098 10.1016/S0140-6736(10)60705-2 20537376 PMC2922041

[B43] Keiser O Anastos K Schechter M Balestre E Myer L Boulle A Bangsberg D Toure H Braitstein P Sprinz E Antiretroviral therapy in resource-limited settings 1996 to 2006: patient characteristics, treatment regimens and monitoring in sub-Saharan Africa, Asia and Latin America Trop Med Int Health 2008 13 870 879 18373510 10.1111/j.1365-3156.2008.02078.x PMC3722496

