# Supplementary material for: Patient- and population-level health consequences of discontinuing antiretroviral therapy in settings with inadequate HIV treatment availability
Source: Cost Eff Resour Alloc. 2012 Sep 19;10:12. doi: 10.1186/1478-7547-10-12 (PMC3502124; doi:10.1186/1478-7547-10-12)
Supplement: Additional file 1 — Supplemental methods and results. [file 1478-7547-10-12-S1.pdf]

Patient- and Population-level Health Consequences of Discontinuing Antiretroviral Therapy in Settings with Inadequate HIV Treatment Availability

Kimmel et al.

Supplementary Appendix

The main objective of this appendix is to provide further details with regard to the methods and results presented in the main text. In particular, we present additional information on the population-level model as well as supplementary sensitivity analysis results.

SUPPLEMENTARY METHODS

Choice of Strategies

A number of public health responses have been implemented in settings with limited resources to treat HIV. These include: later treatment initiation, use of standardized treatment regimens, restricted number of recommended regimens, use of less expensive but potentially more toxic drugs, and less frequent and/or less expensive monitoring of disease progression [1]. One policy response receiving little consideration is ART discontinuation after treatment failure. World Health Organization (WHO) guidelines have recommended continuing treatment in patients who fail the last available regimen [1]. However, some have suggested a discontinuation policy could be considered by decision makers in resource-limited settings to increase treatment access [2].

Individual-level Model

The individual-level model (i.e., the CEPAC-International model) has been well-described [3-9]. Additional details regarding the model — including the model flow chart depicting how hypothetical patients transition through model health states, as well as the mathematical equations used to represent transitions between model health states — are available at http://web2.research.partners.org/cepac.

Population-level Model

Linear programming model

The linear programming model is specified formally in Equation A1, below.

(A1) $$\max_{\{f_{c,i}\}} \sum_{c=1}^{q} \sum_{i=0}^{m} f_{c,i} y_{c,i}$$

where
c = index for incident HIV-infected cohorts that have been detected and diagnosed (c=1,..., q)
i = index for m mutually exclusive treatment strategies (i=0,…, m)
$y_{c,i}$ = life expectancy of persons from incident cohort c and receiving treatment strategy i
$f_{c,i}$ = proportion of individuals from incident cohort c and receiving treatment strategy i

subject to

(A1.a) $\sum_{c=1}^{q}\sum_{i=1}^{m}\pi_{p-c,c,i} f_{c,i} d_c \le x_p \text{ for all } p = 1,\ldots,q$ where

p = index of time periods (p=1, …, q)
$\pi_{n,c,i}$ = proportion receiving treatment in year $n=p–c$ after detection among persons in incident cohort c who are assigned to treatment strategy i
d_c = number of HIV-infected individuals in incident cohort c
x_p = number of available treatment slots in period p

(A1.b) $f_{c,i} \ge 0$ for all c, i

And

(A1.c) $\sum_{i=0}^{m} f_{c,i} = 1, \text{ for all } c$

The population-level, linear programming model consists of the objective function (Equation A1), which seeks to maximize accumulated life-years for multiple cohorts of newly-detected, HIV infected individuals, including those who are not treated; a treatment capacity constraint ensuring that the number of available treatment "slots" in each period is not exceeded by the number of people on active treatment (Equation A1.a); a non-negativity constraint (Equation A1.b) ensuring that implemented levels of each strategy do not fall below 0%; and an implementation constraint (Equation A1.c) ensuring that the sum of the proportion of persons assigned to strategies within each period does not exceed 100%. For the steady state analysis, we assume that $d_c=d$ and $x_p=x$, where d and x are constants (Equation A1.a).

The population-level analysis was implemented in Microsoft Excel using Solver (Frontline Systems) software, which relies on the simplex method [10, 11].

Linear programming model framework

We choose to employ a mathematical programming model not only to provide information on optimal implementation of alternative treatment strategies but also to do so by explicitly accounting for treatment-related resource constraints [11, 12]. The method also holds the capability of implementing mixed solutions (i.e., partial implementation of 2 or more strategies) [13, 14]. We chose to employ a particular class of mathematical programming models, the linear programming model, although we explored the option of employing an integer programming model. In Equation 1a, the decision variable (f_i), which represents the fraction of individuals receiving each treatment strategy, is multiplied by the number of patients seeking treatment (d). This multiplication could lead to fractional numbers of patients receiving treatment. Because individuals are not technically divisible, this suggests use of an integer programming model. However, given the computational complexities associated with solving integer programming problems and given large population sizes, the errors introduced by ignoring this integer constraint are likely to be small [15]. Therefore, we chose a linear programming model for its computational effectiveness and efficiency.

Modeling of treatment capacity

In developing and specifying the model, we chose to model the treatment capacity constraint in terms of treatment slots, defined in terms of numbers of individuals receiving ART annually. Treatment slots were chosen as a proxy for the myriad constraints faced by public sector antiretroviral programs, including financing, human resource capacity, health and social service constraints, and drug, technology, and personnel affordability [16-18]. While recent HIV-related studies have modeled explicitly only funding constraints [19-21], we believe that financing reflects only one dimension of treatment capacity constraints. Therefore, we chose to characterize treatment capacity in terms of a single metric, available treatment "slots", which are limited based on a variety of factors.

Input parameters

The individual-level model produced several projections that were used as inputs to the population-level model. These included: (1) strategy-specific life expectancy estimates for the objective function parameter $y_{c,i}$ (Equation A1), and (2) the number receiving ART annually, which was used to derive strategy-specific annual probability of receiving ART ($\pi_{n,c,i}$ in Equation A1.a) and cohort and strategy-specific annual treatment need (i.e., $\pi_{n,c,i}d$ in Equation A1.a).

Multi-stage Modeling Approach

To highlight the tradeoffs associated with different treatment policies (in this case, discontinuation), this study was conducted using an integrated, or two-stage, modeling approach. While use of integrated modeling approaches has become more visible in the literature, few studies have evaluated HIV treatment policy using this method. Earnshaw and colleagues used output from a Markov model as inputs to a linear programming model in a resource allocation problem applied to diabetes prevention [22]. Brandeau et al. combined an epidemiological model and optimization techniques to inform theory regarding resource allocation considerations in infectious disease epidemic control [23]. Kim et al. linked a series of state-transition models and a binary integer programming model to identify an optimal package of health services in post-reproductive age women undergoing cervical cancer screening [24]. Kim and Goldie used results from a dynamic model as inputs to an individual-level simulation in order to evaluate the cost-effectiveness of vaccination programs against human papillomavirus (HPV) in young girls [25]. Bauch and colleagues approximated dynamic herd immunity effects for a vaccine-preventable pediatric infectious disease and applied these effects to a cohort model used to evaluate a pediatric vaccination program [26]. Finally, Cleary et al. combined a patient-level state-transition model and a linear programming model to assess South African HIV treatment programs, comparing a restricted policy (i.e., 1st-line ART only) and the current standard of care (1st- and 2nd-line ART) [19, 20].

SUPPLEMENTARY RESULTS

Internal Validation of the Individual-level Model

Verification of Equilibrium for the Population-level Model

We conducted an analysis that was restricted to a hypothetical steady state (i.e., both detected cases and treatment capacity were constant). To produce a steady state, the simulation was partitioned into three periods — a burn-in period, analysis period, and censorship period. In the burn-in period, cohorts entered the model and individuals initiated and discontinued (due to a treatment policy or death) ART until the number of individuals entering and exiting treatment from year to year became constant. That is, the burn-in period continued until the model reached equilibrium. Once the model reached equilibrium, we used the analysis period to determine the optimal strategy or combination of strategies that would maximize life expectancy per member of each cohort over a defined timeframe. We also used this period to assess the number of individuals receiving treatment. Finally, rather than terminate the analysis at the end of the analysis period, we included an additional censorship period to account for the life years that would continue to accrue to individuals beyond the analytic timeframe.

We conducted a series of diagnostic evaluations to ensure that the model had reached a steady-state equilibrium. **Figures A1 – A3** show three diagnostic and consistency checks for the population-level linear programming model. In Figure A1, we assessed percent change in life expectancy at the optimum across the periods: burn-in (periods 1–70), analysis (periods 71–80), and censorship (periods 81–100) periods. In the analysis period, we found that the percent change in life expectancy never exceeded 0·01% across the analysis period. In Figure A2, the percent difference at the optimum in the mean number initiating treatment across the analysis period remained relatively constant, never varying by more than 0·0008% between periods. Finally, Figure A3 shows that, beginning in the analysis period, the number of individuals remaining on treatment over time was similar across cohorts.

Sensitivity Analyses

Supplementary selected sensitivity analysis results are shown in **Tables A1 – A4**.

APPENDIX REFERENCES

1. World Health Organization: **Antiretroviral therapy for HIV infection in adults and adolescents: Recommendations for a public health approach. 2010 revision.** 2010.
2. Kimmel AD, Daniels N, Prosser LA: **Decision maker priorities for providing antiretroviral therapy in HIV-infected patients in South Africa: a qualitative assessment.** *Med Decis Making* 2011, **31:**E78.
3. Goldie SJ, Yazdanpanah Y, Losina E, Weinstein MC, Anglaret X, Walensky RP, Hsu HE, Kimmel A, Holmes C, Kaplan JE, Freedberg KA: **Cost-effectiveness of HIV treatment in resource-poor settings--the case of Côte d'Ivoire.** *N Engl J Med* 2006, **355:**1141-1153.
4. Kimmel AD, Weinstein MC, Anglaret X, Goldie SJ, Losina E, Yazdanpanah Y, Messou E, Cotich KL, Walensky RP, Freedberg KA: **Laboratory monitoring to guide switching antiretroviral therapy in resource-limited settings: clinical benefits and cost-effectiveness.** *J Acquir Immune Defic Syndr* 2010, **54:**258-268.
5. Losina E, Touré H, Uhler LM, Anglaret X, Paltiel AD, Balestre E, Walensky RP, Messou E, Weinstein MC, Dabis F, Freedberg KA: **Cost-effectiveness of preventing loss to follow-up in HIV treatment programs: a Côte d'Ivoire appraisal.** *PLoS Med* 2009, **6:**e1000173.
6. Walensky RP, Weinstein MC, Yazdanpanah Y, Losina E, Mercincavage LM, Touré S, Divi N, Anglaret X, Goldie SJ, Freedberg KA: **HIV drug resistance surveillance for prioritizing treatment in resource-limited settings.** *AIDS* 2007, **21:**973-982.
7. Yazdanpanah Y, Losina E, Anglaret X, Goldie SJ, Walensky RP, Weinstein MC, Toure S, Smith HE, Kaplan JE, Freedberg KA: **Clinical impact and cost-effectiveness of co-trimoxazole prophylaxis in patients with HIV/AIDS in Cote d'Ivoire: a trial-based analysis.** *AIDS* 2005, **19:**1299-1308.
8. Walensky RP, Wolf LL, Wood R, Fofana MO, Freedberg KA, Martinson NA, Paltiel AD, Anglaret X, Weinstein MC, Losina E: **When to start antiretroviral therapy in resource-limited settings.** *Ann Intern Med* 2009, **151:**157-166.
9. Walensky RP, Wood R, Ciaranello AL, Paltiel AD, Lorenzana SB, Anglaret X, Stoler AW, Freedberg KA: **Scaling up the 2010 World Health Organization HIV Treatment Guidelines in resource-limited settings: a model-based analysis.** *PLoS Med,* **7:**e1000382.
10. Fylstra D, Lasdon L, Watson J, al. e: **Design and use of the Microsoft Excel Solver.** *Interfaces* 1998, **28:**29-55.
11. Hillier FS, Lieberman GJ: *Introduction to operations research.* 8th edn. Boston: McGraw-Hill Higher Education; 2005.
12. Arrow KJ, Intriligator MD: *Handbook of mathematical economics.* Amsterdam ; New York; 1981.
13. Birch S, Gafni A: **Cost effectiveness/utility analyses. Do current decision rules lead us to where we want to be?** *J Health Econ* 1992, **11:**279-296.
14. Stinnett AA, Paltiel AD: **Mathematical programming for the efficient allocation of health care resources.** *J Health Econ* 1996, **15:**641-653.
15. Garey MR, Johnson DS: *Computers and intractability : a guide to the theory of NP-completeness.* San Francisco: W. H. Freeman; 1979.

16. Loewenson R, McCoy D: **Access to antiretroviral treatment in Africa.** *BMJ* 2004, **328:**241-242.
17. McCoy D, Chopra M, Loewenson R, Aitken JM, Ngulube T, Muula A, Ray S, Kureyi T, Ijumba P, Rowson M: **Expanding access to antiretroviral therapy in sub-saharan Africa: avoiding the pitfalls and dangers, capitalizing on the opportunities.** *Am J Public Health* 2005, **95:**18-22.
18. Schneider H, Blaauw D, Gilson L, Chabikuli N, Goudge J: **Health systems and access to antiretroviral drugs for HIV in Southern Africa: service delivery and human resources challenges.** *Reprod Health Matters* 2006, **14:**12-23.
19. Cleary S, Mooney G, McIntyre D: **Equity and efficiency in HIV-treatment in South Africa: the contribution of mathematical programming to priority setting.** *Health Econ* 2009.
20. Cleary SM, McIntyre D, Boulle AM: **Assessing efficiency and costs of scaling up HIV treatment.** *AIDS* 2008, **22 Suppl 1:**S35-42.
21. Earnshaw SR, Hicks K, Richter A, Honeycutt A: **A linear programming model for allocating HIV prevention funds with state agencies: a pilot study.** *Health Care Manag Sci* 2007, **10:**239-252.
22. Earnshaw SR, Richter A, Sorensen SW, Hoerger TJ, Hicks KA, Engelgau M, Thompson T, Narayan KM, Williamson DF, Gregg E, Zhang P: **Optimal allocation of resources across four interventions for type 2 diabetes.** *Med Decis Making* 2002, **22:**S80-91.
23. Brandeau ML, Zaric GS, Richter A: **Resource allocation for control of infectious diseases in multiple independent populations: beyond cost-effectiveness analysis.** *J Health Econ* 2003, **22:**575-598.
24. Kim JJ, Salomon JA, Weinstein MC, Goldie SJ: **Packaging health services when resources are limited: the example of a cervical cancer screening visit.** *PLoS Med* 2006, **3:**e434.
25. Kim JJ, Goldie SJ: **Health and economic implications of HPV vaccination in the United States.** *N Engl J Med* 2008, **359:**821-832.
26. Bauch CT, Anonychuk AM, Effelterre TV, Pham BZ, Merid MF: **Incorporating Herd Immunity Effects into Cohort Models of Vaccine Cost-Effectiveness.** *Med Decis Making* 2009.
27. Losina E, Yazdanpanah Y, Deuffic-Burban S, Wang B, Wolf LL, Messou E, Gabillard D, Seyler C, Freedberg KA, Anglaret X: **The independent effect of highly active antiretroviral therapy on severe opportunistic disease incidence and mortality in HIV-infected adults in Côte d'Ivoire.** *Antivir Ther* 2007, **12:**543-551.
28. Tuboi SH, Brinkhof MW, Egger M, Stone RA, Braitstein P, Nash D, Sprinz E, Dabis F, Harrison LH, Schechter M: **Discordant responses to potent antiretroviral treatment in previously naive HIV-1-infected adults initiating treatment in resource-constrained countries: the antiretroviral therapy in low-income countries (ART-LINC) collaboration.** *J Acquir Immune Defic Syndr* 2007, **45:**52-59.

Table A1. Selected sensitivity analysis results: treatment policy and management

Strategy*	Treated Individuals Only: Life Expectancy (Years)	Treated Individuals Only: Mean Time on Treatment (Years)	Treated and Untreated Individuals†: Mean Number Initiating Treatment Annually	Treated and Untreated Individuals†: Life Expectancy (Years)	Treated and Untreated Individuals†: Total Life-Years (Years)	Treated and Untreated Individuals†: Mean Annual Treatment Coverage‡ (%)
**Number of available ART regimens§**						
One ART regimen available only						
Status Quo	7·3	6·2	7,090	3·5	530,000	29·4
Alternative	6·0	4·1	11,110	3·8	572,000	46·1
Three ART regimens available						
Status Quo	9·5	8·0	5,420	3·7	548,000	22·5
Alternative	9·0	7·4	5,960	3·7	556,000	24·7
**ART initiation criteria§**						
CD4 count <200 cells/μL						
Status Quo	8·5	7·0	5,250	3·6	543,000	25·6
Alternative	7·7	6·0	6,230	3·7	557,000	30·4
**Patient-level treatment monitoring**						
+HIV RNA monitoring¶						
Status Quo	8·7	7·3	5,950	3·6	542,000	24·7
Alternative	7·6	5·4	8,230	3·9	580,000	34·2
CD4 count decrease 25%‖						
Status Quo	9·0	7·6	5,750	3·6	545,000	23·9
Alternative	8·5	6·7	6,590	3·7	561,000	27·4

Abbreviations: ART = antiretroviral therapy.

* In the Status Quo, antiretroviral therapy (ART) is never discontinued. In the Alternative strategy, ART is discontinued when second-line ART failure is observed. In the base case, ART failure is defined as a 50% decrease in peak on-treatment CD4 count, CD4 count <100 cells/μL, CD4 count below pre-ART nadir, or a WHO stage III/IV event, excluding tuberculosis and severe bacterial infections [1]. On average, individuals who received no treatment lived approximately 1·9 years.

† Results are for a 5-year analytic time horizon for a cohort of 30,000 newly detected infected individuals entering care annually.

‡ Treatment coverage is defined as the ratio of the number receiving treatment annually to the number qualifying for treatment annually.

§ In the base case, patients received two sequential ART regimens. ART was initiated at CD4 count <350 cells/μL or a WHO stage III/IV event.

¶ In this sensitivity analysis, both semiannual HIV RNA monitoring and virologic antiretroviral failure criteria (>500 copies/mL) are added to the base case monitoring and antiretroviral failure detection assumptions.

‖ In this sensitivity analysis, ART failure is defined defined as a 25% decrease in peak on-treatment CD4 count, CD4 count <100 cells/μL, CD4 count below pre-ART nadir, or a WHO stage III/IV event, excluding tuberculosis and severe bacterial infections [1].

Table A2. Selected sensitivity analysis results: loss from and return to treatment

Strategy*	Treated Individuals Only: Life Expectancy (Years)	Treated Individuals Only: Mean Time on Treatment (Years)	Treated and Untreated Individuals†: Mean Number Initiating Treatment Annually	Treated and Untreated Individuals†: Life Expectancy (Years)	Treated and Untreated Individuals†: Total Life-Years (Years)	Treated and Untreated Individuals†: Mean Annual Treatment Coverage‡ (%)
**18-month loss to follow-up§**						
10% increase						
Status Quo	8·5	7·1	6,170	3·6	550,000	25·9
Alternative	7·8	6·0	7,300	3·7	560,000	30·6
10% decrease						
Status Quo	9·1	7·9	5,590	3·6	541,000	23·0
Alternative	8·3	6·6	6,680	3·7	556,000	27·4
No loss to follow-up						
Status Quo	14·7	14·6	3,140	3·4	517,000	11·8
Alternative	12·5	11·2	4,130	3·6	537,000	15·4
**Return to care after becoming lost§**						
10% increase						
Status Quo	8·9	7·5	5,830	3·6	543,000	24·2
Alternative	8·1	6·4	6,940	3·7	559,000	28·8
10% decrease						
Status Quo	8·7	7·3	5,930	3·6	543,000	24·7
Alternative	8·0	6·2	7,040	3·7	558,000	29·3
No return to care						
Status Quo	8·0	6·5	6,590	3·6	540,000	27·3
Alternative	7·4	5·7	7,650	3·7	553,000	31·8

* In the Status Quo, antiretroviral therapy (ART) is never discontinued. In the Alternative strategy, ART is discontinued when second-line ART failure is observed. In the base case, ART failure is defined as a 50% decrease in peak on-treatment CD4 count, CD4 count <100 cells/μL, CD4 count below pre-ART nadir, or a WHO stage III/IV event, excluding tuberculosis and severe bacterial infections [1]. On average, individuals who received no treatment lived approximately 1·9 years.

† Results are for a 5-year analytic time horizon for a cohort of 30,000 newly detected infected individuals entering care annually.

‡ Treatment coverage is defined as the ratio of the number receiving treatment annually to the number qualifying for treatment annually.

§ In the base case, 15% were lost from care by 18 months. We assumed that 50% of those experiencing a WHO stage III/IV event after becoming lost would return to treatment and care.

Table A3. Selected sensitivity analysis results: antiretroviral efficacy and response to ART

	Treated Individuals Only		Treated and Untreated Individuals†			
Strategy*	Life Expectancy (Years)	Mean Time on Treatment (Years)	Mean Number Initiating Treatment Annually	Life Expectancy (Years)	Total Life-Years (Years)	Mean Annual Treatment Coverage‡ (%)
**2nd-line ART effectiveness§**						
10% increase						
Status Quo	8.9	7.6	5,770	3.6	544,000	24.0
Alternative	8.2	6.4	6,830	3.7	559,000	28.4
10% decrease						
Status Quo	8·6	7·3	6,000	3.6	542,000	24.9
Alternative	7·9	6·2	7,150	3.7	558,000	29.7
**"Late" 2nd-line ART failure§**						
10% increase						
Status Quo	8·7	7·4	5,930	3·6	543,000	24·6
Alternative	8·0	6·3	7,030	3·7	558,000	29·2
10% decrease						
Status Quo	8.8	7.5	5,840	3.6	544,000	24.3
Alternative	8.1	6.4	6,930	3.7	558,000	28.8
**Independent effect of ART on mortality¶**						
10% increase						
Status Quo	8·9	7·6	5,790	3·6	544,000	24·0
Alternative	8·2	6·4	6,920	3·7	559,000	28·6
10% decrease						
Status Quo	8·6	7·3	5,960	3·6	542,000	24·9
Alternative	7·9	6·2	7,060	3·7	557,000	29·5
No independent effect						
Status Quo	4·5	3·7	10,590	3·3	490,000	52·4
Alternative	4·4	3·5	11,210	3·3	498,000	55·5
**Discordant responses among virologically suppressed‖**						
*19.1% discordant responses***						
Status Quo	8·3	7·0	6,220	3·6	539,000	26·0
Alternative	7·4	5·7	7,720	3·7	556,000	32·2
10% discordant responses						
Status Quo	8·6	7·3	5,990	3·6	542,000	24·9

Alternative	7·8	6·1	7,220	3·7	558,000	30·1
No discordant responses						
Status Quo	9·0	7·6	5,780	3·6	544,000	24·0
Alternative	8·3	6·5	6,760	3·7	559,000	28·0

Abbreviations: ART = antiretroviral therapy.

* In the Status Quo, antiretroviral therapy (ART) is never discontinued. In the Alternative strategy, ART is discontinued when second-line ART failure is observed. In the base case, ART failure is defined as a 50% decrease in peak on-treatment CD4 count, CD4 count <100 cells/μL, CD4 count below pre-ART nadir, or a WHO stage III/IV event, excluding tuberculosis and severe bacterial infections [1]. On average, individuals who received no treatment lived approximately 1·9 years.

† Results are for a 5-year analytic time horizon for a cohort of 30,000 newly detected infected individuals entering care annually.

‡ Treatment coverage is defined as the ratio of the number receiving treatment annually to the number qualifying for treatment annually.

§ Second-line antiretroviral effectiveness refers to 24-week HIV RNA suppression after 2nd-line ART initiation. "Late" 2nd-line ART failure refers to ART failure after six months on 2nd-line ART.

¶ Patients with virologic failure but who remain on ART have an independent reduction in AIDS-related mortality compared to those not receiving ART [27].

‖ In the base case, five percent of virologically suppressed patients on ART experienced a discordant response, or no immunologic response to ART.

** Based on data reported in Tuboi *JAIDS* 2007 [28].

Table A4. Selected sensitivity analysis results: treatment capacity

Strategy*	Treated Individuals Only: Life Expectancy (Years)	Treated Individuals Only: Mean Time on Treatment (Years)	Treated and Untreated Individuals†: Mean Number Initiating Treatment Annually	Treated and Untreated Individuals†: Life Expectancy (Years)	Treated and Untreated Individuals†: Total Life-Years (Years)	Treated and Untreated Individuals†: Mean Annual Treatment Coverage‡ (%)
**Treatment capacity exceeds treatment demand§**						
10,000 newly detected per year; 50,000 available slots						
Status Quo	8.8	7.4	5,880	7.0	348,000	73.3
Alternative	8.1	6.3	6,980	7.3	363,000	86.9
**Treatment capacity equals treatment demand §**						
50,000 newly detected per year; 50,000 available slots						
Status Quo	8.8	7.4	5,880	3.0	738,000	14.7
Alternative	8.1	6.3	6,980	3.0	753,000	17.4
**Treatment demand exceeds treatment capacity§**						
125,000 newly detected per year; 50,000 available slots						
Status Quo	8.8	7.4	5,880	2.4	1,470,000	5.9
Alternative	8.1	6.3	6,980	2.4	1,485,000	7.0

* In the Status Quo, antiretroviral therapy (ART) is never discontinued. In the Alternative strategy, ART is discontinued when second-line ART failure is observed. In the base case, ART failure is defined as a 50% decrease in peak on-treatment CD4 count, CD4 count <100 cells/µL, CD4 count below pre-ART nadir, or a WHO stage III/IV event, excluding tuberculosis and severe bacterial infections [1]. On average, individuals who received no treatment lived approximately 1·9 years.

† Results are for a 5-year analytic time horizon for a cohort of 30,000 newly detected infected individuals entering care annually.

‡ Treatment coverage is defined as the ratio of the number receiving treatment annually to the number qualifying for treatment annually.

§ In the base case, there were 30,000 newly detected, HIV-infected patients each year and 50,000 total treatment slots available at any one time.

APPENDIX LEGENDS

Figure A1. Percent change in life expectancy across cohorts, by period of analysis
The *x*-axis shows the number of cohorts assessed and the *y*-axis shows the percent change in life expectancy. As the number of cohorts evaluated increased, the percent change in life expectancy decreased. #: number.

Figure A2. Average number initiating antiretroviral therapy over time, by period of analysis
The *x*-axis shows time (in years); the *y*-axis shows the number initiating antiretroviral therapy. Over time, and as the number of cohorts evaluated increased, the number initiating antiretroviral therapy annually became relatively constant. #: number; ART: antiretroviral therapy.

Figure A3. Number of individuals remaining on treatment in previous 10 years, by time period
The *x*-axis shows the incident cohort at time *t*; the *y*-axis shows the number remaining on treatment in each incident cohort. All of the lines in the figure are overlapping, which suggests that the number initiating and remaining in care in each analysis period year is constant.
#: number.

Figure A1

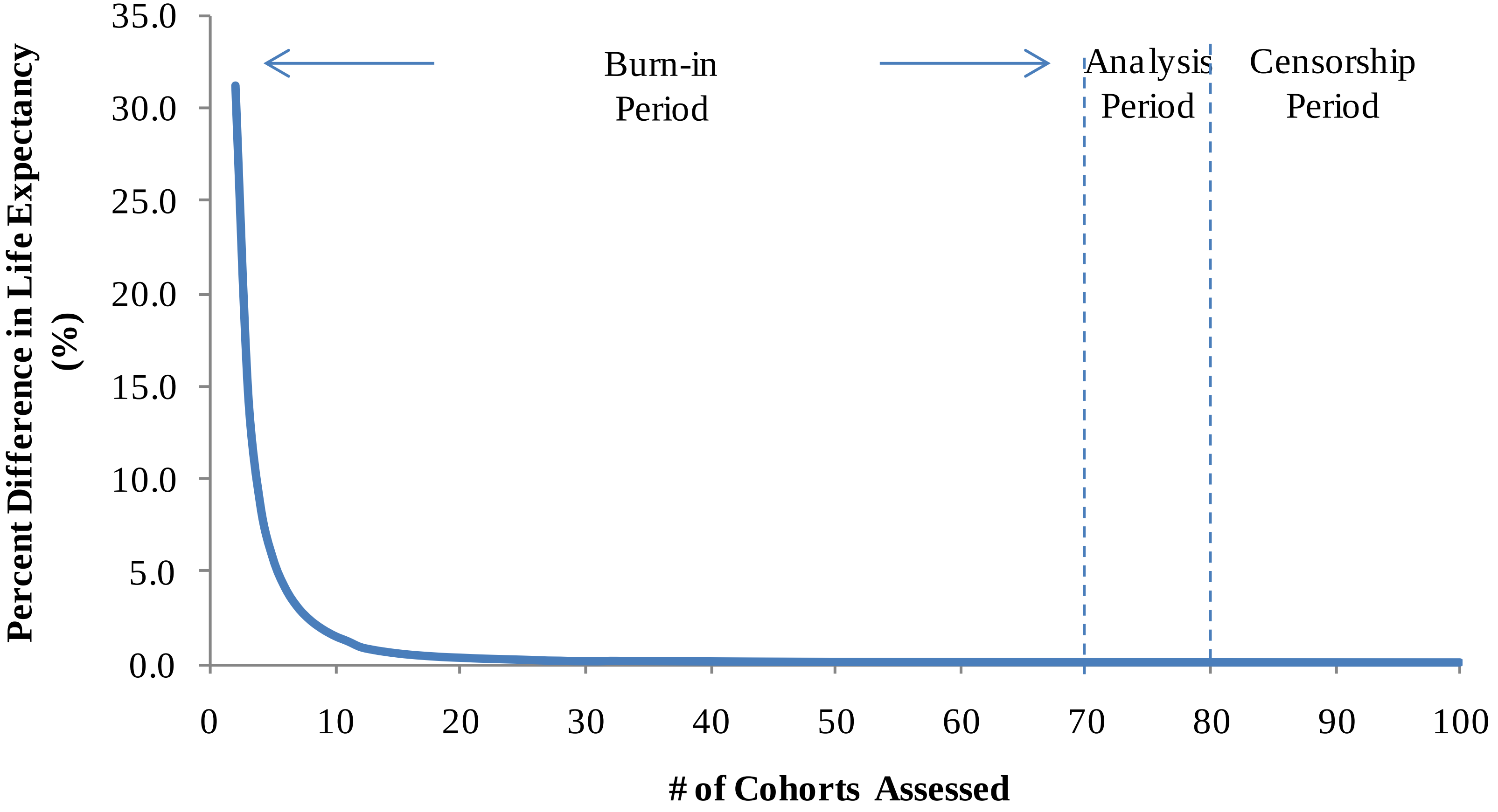
Percent Difference in Life Expectancy (%)
35.0
30.0
25.0
20.0
15.0
10.0
5.0
0.0
0
10
20
30
40
50
60
70
80
90
100
Burn-in Period
Analysis Period
Censorship Period
# of Cohorts Assessed

Figure A2

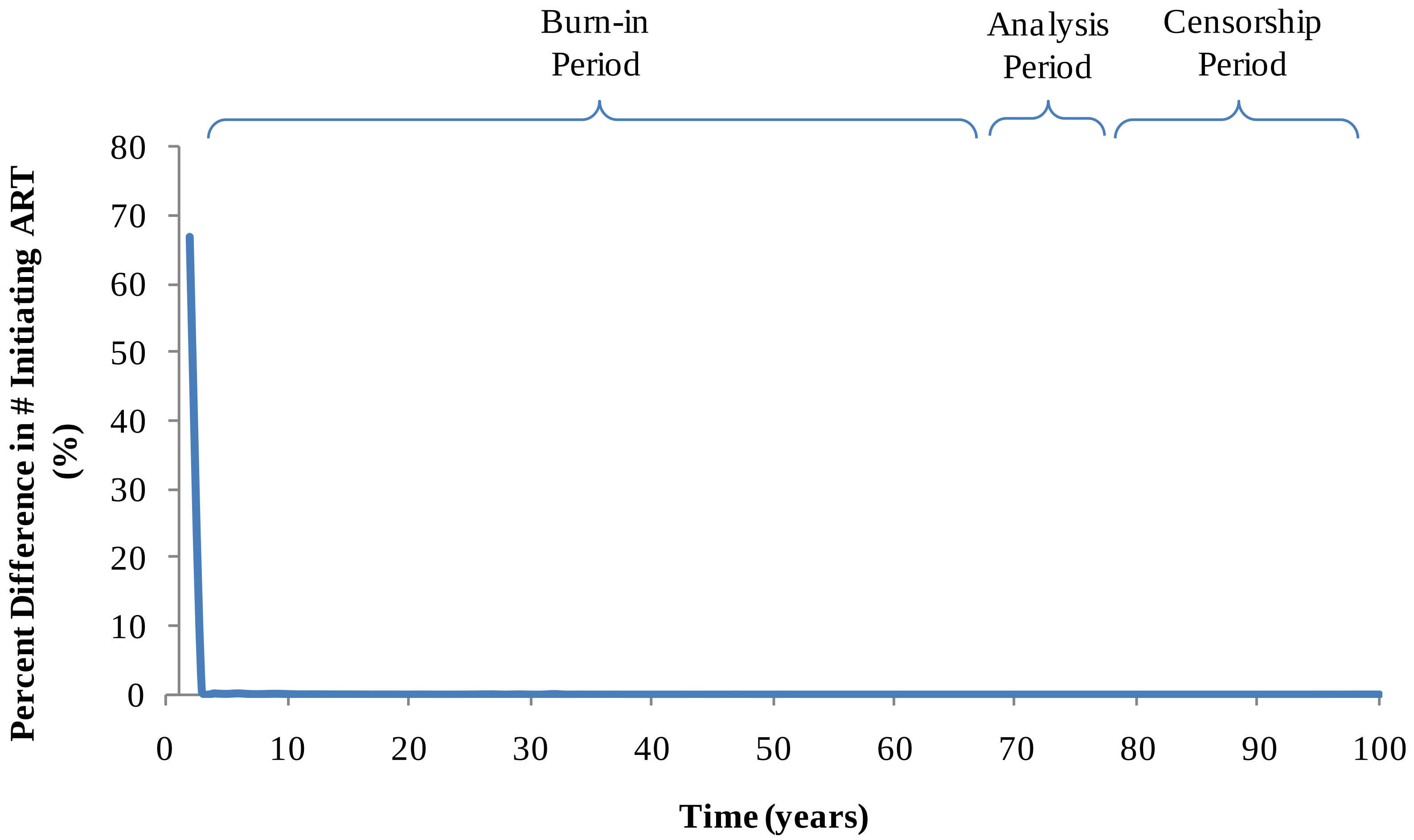

Burn-in
Period
Analysis
Period
Censorship
Period
Percent Difference in # Initiating ART
(%)
80
70
60
50
40
30
20
10
0
0
10
20
30
40
50
60
70
80
90
100
Time (years)

Figure A3

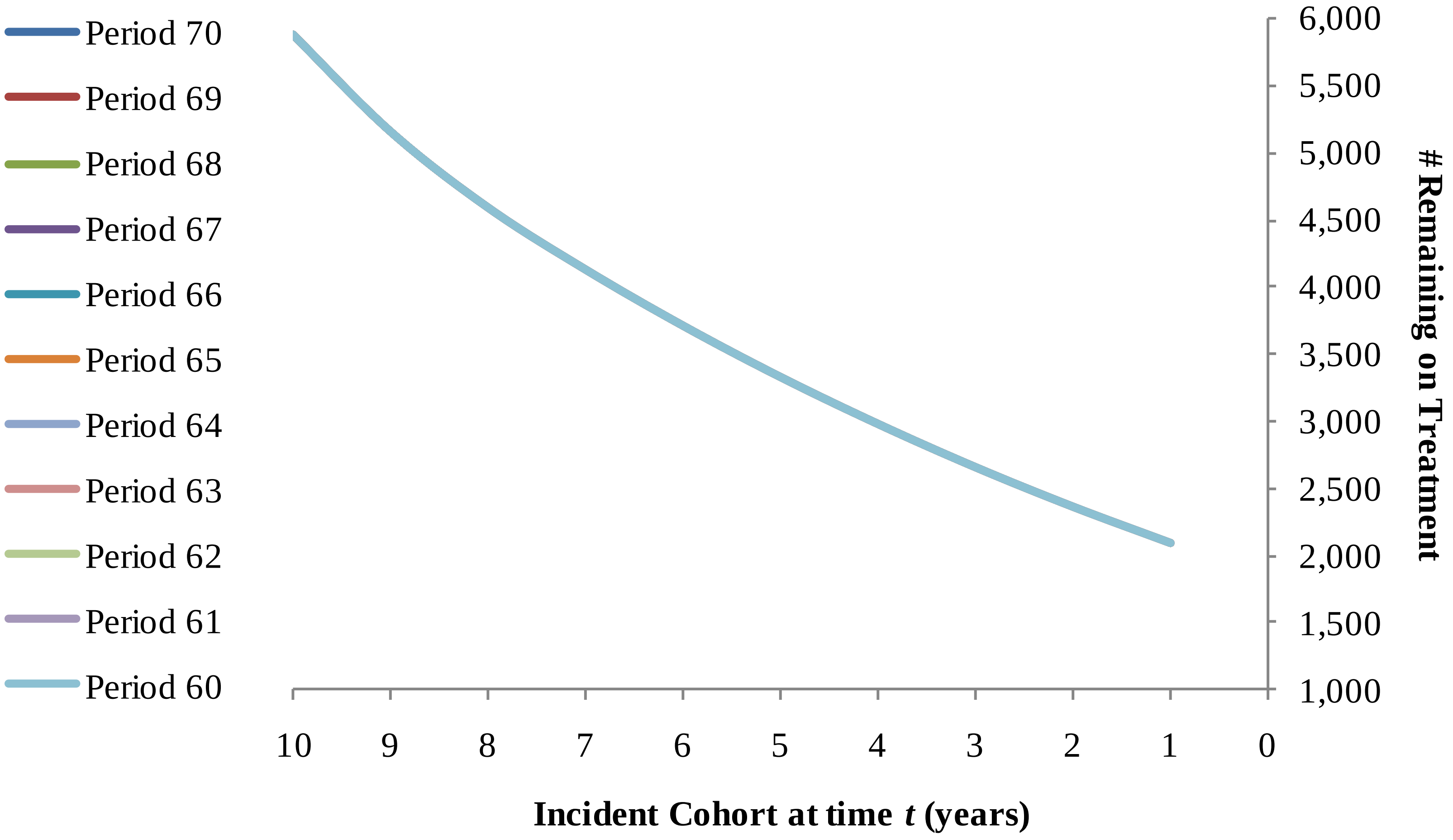

Period 70
Period 69
Period 68
Period 67
Period 66
Period 65
Period 64
Period 63
Period 62
Period 61
Period 60
6,000
5,500
5,000
4,500
4,000
3,500
3,000
2,500
2,000
1,500
1,000
Remaining on Treatment
10
9
8
7
6
5
4
3
2
1
0
Incident Cohort at time *t* (years)
